# PP75 Empowering Laypeople In Health Technology Assessment Through Gamification: The PARTICIPA SUS/ATS Project

**DOI:** 10.1017/S0266462325102936

**Published:** 2025-12-29

**Authors:** Quenia Cristina Dias Morais, Marcelo Goulart, Dominique Spada, Milene Costa, Bruno Barros, Andressa Braga, Marisa Santos

## Abstract

**Introduction:**

Patient involvement is a cornerstone of the Brazilian Unified Health System (SUS), legitimizing decisions on the reimbursement of new health technologies. However, the complexity of these processes hinders public engagement. To overcome this, the Participa SUS/ATS project developed a gamified digital platform to educate laypeople about health technology assessment (HTA), empowering them with knowledge to engage effectively in public consultations.

**Methods:**

The platform has individualized access and comprises ten modules of educational videos and interactive games. Joana, a fictional SUS user, embarks on a journey reflecting common patient experiences, learning about the health system, HTA, and technology reimbursement processes. Content was informed by research into challenges faced by patient associations and validated through online questionnaires with pilot users. Videos used simplified language, illustrations, and Brazilian Sign Language (Libras) to enhance accessibility. Games reinforced learning by aligning with Joana’s narrative. Upon completing modules, users receive certificates. The approach prioritized inclusivity, creating an engaging and relatable environment for diverse learning needs.

**Results:**

Gamification, centered on Joana’s relatable journey, effectively engaged SUS users. Participants reported improved understanding of HTA concepts. Evaluative games reinforced knowledge, and over 300 certificates were issued upon module completion, marking participant progress. Joana’s narrative resonated deeply, fostering accessibility and identification. Inclusive features, such as Libras, simplified language, and illustrations, broadened engagement and reflected Brazil’s socioeconomic and cultural diversity. The project demonstrated that narrative-driven gamification is a powerful tool for translating complex technical topics into practical knowledge, empowering lay audiences to participate actively in decision-making processes.

**Conclusions:**

The Participa SUS/ATS platform demonstrates that gamification, combined with relatable narratives, can demystify technical topics like HTA, fostering informed public participation. This initiative enhanced social participation in the SUS, empowering users to contribute actively and knowledgeably to decision-making. This model can be adapted to other topics, broadening its impact by promoting citizenship and fostering engagement in public health initiatives.

